# Assessing Fidelity to HIV Index Case Testing Protocols in Malawian Clinical Settings: Results From a Cluster Randomized Controlled Trial

**DOI:** 10.1002/jia2.70185

**Published:** 2026-10-08

**Authors:** Tapiwa A. Tembo, Nora E. Rosenberg, Katie R. Mollan, Mike J. Chitani, Mtisunge Mphande, Jiayu Wang, Victor Mwapasa, Katherine Simon, Saeed Ahmed, Maria H. Kim, Linda‐Gail Bekker

**Affiliations:** ^1^ Baylor College of Medicine Children's Foundation Lilongwe Malawi; ^2^ Gillings School of Global Public Health University of North Carolina at Chapel Hill Chapel Hill North Carolina USA; ^3^ School of Medicine University of North Carolina at Chapel Hill Chapel Hill North Carolina USA; ^4^ Kamuzu University of Health Sciences Blantyre Malawi; ^5^ Department of Pediatrics Baylor College of Medicine Houston Texas USA; ^6^ The Desmond Tutu HIV Centre Institute of Infectious Disease and Molecular Medicine Faculty of Health Sciences University of Cape Town Cape Town South Africa

**Keywords:** blended learning, CHWs, digital training, fidelity, HIV, index case testing, lay health workers, LMIC, partner notification

## Abstract

**Introduction:**

HIV index case testing is an evidence‐based practice in which health care workers (HCWs) counsel people living with HIV (index clients) to offer HIV testing services to their sexual partners and biological children (contacts). For index case testing to be effective, it is important that index‐ and contact‐client counselling protocols are implemented as intended (fidelity). We evaluated whether a digital training implementation strategy improved HCWs’ fidelity to these counselling protocols.

**Methods:**

We conducted a cluster‐randomized controlled trial at health facilities (clusters) in Southeastern Malawi. Primary study follow‐up was from October 2022 to September 2023. Thirty‐three clusters were randomized 1:2 to the Enhanced (*n* = 11) and Standard (*n* = 22) arms. HCWs in both arms received brief centralized in‐person training on processes and protocols for index case testing. HCWs in the Enhanced arm also received decentralized tablet‐guided digital training through 20 synchronous and asynchronous sessions. In all 33 clusters, a selection of index and contact client encounters were audio‐recorded in the year after the digital training. We applied a 15‐item fidelity checklist to assess counselling quality and adherence to index case testing protocols. Audio recordings were coded independently by two assessors blinded to the study arm. Fidelity scores ranged from 0% to 100%; higher scores indicate better protocol compliance. To compare fidelity scores between the Enhanced and Standard arms, we estimated a mean difference and 95% confidence interval (CI), using generalized estimating equations to account for clustered data.

**Results:**

We audio‐recorded 341 index client‐HCW and 194 contact client‐HCW encounters. The mean fidelity score for index client‐HCW encounters was 66.3% (CI: 62.8%−69.7%) in the Enhanced arm and 45.7% (CI: 42.7%−48.6%) in the Standard arm (Mean Difference 20.6%; CI: 16.1%, 25.2%, *p*<0.001). The mean fidelity score for contact client‐HCW encounters was 69.0% (CI: 63.8%−74.1%) in the Enhanced arm and 57.4% (CI: 55.5%−59.3%) in the Standard arm (Mean Difference 11.5%; CI: 6.1%, 17.1%, *p*<0.001).

**Conclusions:**

The digital training substantially improved HCW fidelity to index and contact client protocols. A decentralized digital training can be used to improve HCW counselling behaviour. Future research should explore the methods of assessing fidelity in trials of complex digital training interventions in other settings.

## 1 Introduction

Globally, an estimated 40.8 million people were living with HIV (PLHIV) in 2024, primarily from low‐ and middle‐income countries (LMICs) [1]. Approaches are needed to find the remaining 5.3 million undiagnosed PLHIV and link them to antiretroviral therapy (ART) in support of UNAIDS’ 95–95–95 goals by 2030 [2]. Index case testing is an evidence‐based intervention in which the sex partners, biological children and household members of PLHIV are notified of their exposure to HIV and offered testing. Index case testing efficiently leads to new diagnoses of PLHIV [3, 4]. WHO guidelines recommend index case testing approaches [5] as they are safe, effective and efficient for reaching HIV‐exposed individuals and linkage to care [3, 6, 7, 8, 9].

For index case testing services to be effective, fidelity of the services must be maintained [10, 11]. Fidelity refers to the extent to which core components of an intervention are delivered as intended [12]. For index case testing, it consists of a health worker interviewing PLHIV about their contacts and discussing referral options for HIV testing services [13]. It requires approaching these encounters in a client‐centred, voluntary, confidential and non‐judgemental manner. Our formative work [14] revealed that health worker fidelity to these principles and protocols was lacking, a finding mirrored in similar contexts [15].

In‐service training for health workers is frequently used to improve healthcare service delivery [16] and may improve fidelity to index case testing procedures. However, centralized in‐person training strategies are expensive, time‐consuming and disruptive to the provision of healthcare [17, 18, 19]. Decentralized, digital‐based training may be more efficient and less disruptive. We designed and pilot‐tested a digital training to improve fidelity, with promising preliminary results [14]. We then evaluated the digital training in a large‐scale cluster‐randomized controlled trial (CRCT) [20]. The CRCT improved HIV index case testing indicators, including rates of contact client elicitation, testing and diagnosis [21]. This analysis examines a mechanistic outcome: fidelity.

This analysis focuses on the impact of digital training on health workers’ fidelity to index and contact client counselling protocols. We hypothesized that the digital training implementation strategy would improve health workers’ fidelity to index case testing protocols in a real‐world setting.

## 2 Methods

### 2.1 Study Setting

The study was conducted in Malawi, a country with an HIV prevalence of 8.9% [22]. To manage the HIV burden, Malawi task‐shifted the provision of HIV testing services, including index case testing, to lay cadres.

This analysis was part of the PRACTICE study (Package of Resources for Assisted Contact Tracing: Implementation, Cost, and Effectiveness) [20]. PRACTICE was conducted at 14 health facilities in Balaka and 20 health facilities in Machinga. The 34 facilities were supported by the Tingathe Program, a PEPFAR‐funded affiliate of Baylor College of Medicine Children's Foundation Malawi. In partnership with the Malawi Ministry of Health (MOH), Tingathe provided technical assistance and direct service provision, including HIV index testing services by lay health care workers (HCWs) [23] according to Malawi MOH guidelines.

Lay HCWs were expected to identify index clients (PLHIV who were newly diagnosed or already on ART), provide counselling and elicit sexual partners, biological children, and members of their social networks (contact clients) in need of HIV testing services. Index clients were then supported to recruit their contacts for HIV testing services through a variety of tracing modalities. Contact clients were then offered HIV testing services, and those newly diagnosed as living with HIV were linked to treatment and care. Consenting clients who failed to report to the facility for testing were traced through phone or physical tracing in accordance with MOH and programme procedures.

### 2.2 Study Design

PRACTICE was a CRCT with facilities corresponding to clusters [20]. Two neighbouring facilities were combined to create one cluster for purposes of 1:2 randomization. The 33 clusters were randomized to a 1:2 ratio to standard of care plus the digital training implementation strategy (Enhanced arm, *n* = 11) versus standard of care training (Standard arm, *n* = 22). The PRACTICE study had three sets of outcomes: (1) implementation; (2) clinical effectiveness; and (3) cost‐effectiveness. The study described here focused on fidelity, the primary implementation outcome.

The standard training was offered in October 2021. The digital training implementation strategy was delivered in August−September 2022, and the primary follow‐up period for this analysis was October 2022−September 2023.

### 2.3 Implementation Strategies

Both arms received a one‐time, centralized synchronous facilitator‐delivered index case testing training before the start of the trial. The Enhanced arm additionally received a digital training implementation strategy that included 8 h of individual‐level teaching and modelling (five sessions), 14 h of small‐group practice (eight sessions), virtual feedback (one session) and a series of monthly 2‐h continuous quality improvement activities (six sessions). The full details of the implementation strategies are described previously [20]. The development of the digital training was guided by formative work [14], the social cognitive theory [24] and Ericsson's theory of expertise [25]. The digital training aimed to provide knowledge on principles of index case testing, model index and contact clients counselling, allow HCWs to practice counselling and receive individual feedback.

### 2.4 Study Population

Three populations were recruited for PRACTICE: HCWs, index clients and contact clients [20].

HCWs were recruited prior to the enhanced training from May to July 2022. HCWs from the 33 clusters were recruited if they were aged 18 years or older, working full‐time at the facility and tasked with providing index case testing services. All health workers at the facility who met the eligibility criteria were offered the opportunity to provide informed consent and enrol in the study. HCWs who were enrolled in the study participated in the following activities, including: (1) three surveys (before the digital training, just after the training and 1 year after the training); and (2) recorded counselling sessions with a subset of index and contact clients in the subsequent year. Their participation lasted for 2 years.

Index and contact clients were recruited from all 33 clusters between October 2022 and September 2023. Eligibility criteria included age 15 years or older and being an index client, contact client or the parent/guardian of a paediatric index client or contact client. The research team visited each facility at least one time each month and enrolled eligible clients willing to join the study on that day. Index and contact clients recruited were adults (≥18 years) and paediatric clients (below 18 years). A balance of index and contact clients were recruited from all facilities. Index and contact clients recruited in the trial participated in audio‐recordings of their counselling sessions and one‐time exit interviews. Clients’ data were abstracted from facility registers. Their participation lasted for that single visit.

### 2.5 Data Collection Procedures

Index clients were recruited from two settings at the facility: the room where HIV testing services were provided and the ART clinic room. Contact clients were recruited from the facility or community setting. The abstracted data were entered into SurveyCTO (Dobility, Inc., MA).

### 2.6 Study Outcomes and Measurements

Our primary outcome for this analysis was HCWs fidelity to index case testing counselling. There were two fidelity outcomes: (1) fidelity score for index clients; and (2) fidelity score for contact clients.

Fidelity to index case testing protocols was evaluated by considering both adherence to core elements and quality of delivery as described by Proctor et al. [12]. Adherence was conceptualized as the extent to which intervention components were implemented as designed. The quality of intervention delivery indicated the way or how the index case testing counselling was being delivered [12, 26].

We conducted a fidelity assessment to evaluate how well lay HCWs adhered to the index case testing counselling procedures. These assessments were conducted through audio‐recording of index case testing counselling sessions between health workers and index clients or health workers and contact clients. Research staff audio‐recorded the lay HCW‐client encounters. Fidelity assessments involved lay HCWs conducting index case testing counselling sessions with real index clients at the facility and contact clients at the facility or in the community. The counselling sessions were conducted in Chichewa. A minimum of four index client‐HCW and four contact client‐HCW encounters were selected through systematic sampling from each cluster and audio‐recorded. For all fidelity assessments, both the client and HCW had provided informed consent to participate in the study.

Fidelity assessments were conducted with enrolled index or contact clients. Fidelity assessments were conducted using two 15‐item checklists—one for encounters with index clients and one for encounters with contact clients (Supplementary Material 1). We used the same 15‐item fidelity checklists based on the items in the training to assess adherence and quality. Adherence to index client procedures included introducing the session, obtaining verbal consent, eliciting all potential contacts, assessing index client safety, discussing options for recruiting each contact, helping the index clients select an appropriate contact method, discussing the logistics of the methods selected and summarizing the action points. Adherence to contact client procedures were similar: introducing the session, obtaining verbal consent, discussing HIV testing history and linkage to prevention and treatment, discussing logistics of the HIV testing method selected, conducting HIV testing and treatment or prevention referral if needed, providing index counselling if the contact client is also an index, and summarizing the action points.

For both index and contact clients, the quality of counselling was evaluated as the extent to which it was client‐centred, non‐judgemental, culturally appropriate, and had a natural order and flow.

The fidelity checklists were piloted to determine the utility of the tools in conducting fidelity assessments. To assess the content of the tools (the extent to which the checklists accurately assess what they intended to measure) [27], Tingathe index case testing training managers qualitatively reviewed the items of the checklists to ensure they captured key concepts of index case testing and provided feedback.

Scoring criteria were developed, and two assessors (research staff) were trained on these criteria to ensure consistency prior to conducting assessments. For each of the quality items, a set of positive and negative examples were provided to support operationalization. For example, for non‐judgemental counselling, examples of gentle and harsh tones of voice, appropriate and inappropriate word choices, and the presence or absence of empathic statements were provided. For natural order and flow, examples of smooth and awkward responses were provided. We then reviewed the first 10 coded files from both assessors to identify and reconcile discrepancies and to achieve a common understanding through further training. Discrepancies were resolved through discussion following consensus methods.

Both assessors then coded all fidelity assessments. Assessors were blinded to the study arm. The assessors were expected to listen to the audio file at least twice before providing a score. For each audio recording, assessors provided a score of needs improvement (0), satisfactory (1) or excellent (2) on each of the 15 items. There was a maximum score of 30 points, which was then converted to a percentage (0%–100%). Higher scores indicated higher fidelity. Fidelity checklists were first recorded on paper forms and then entered into SurveyCTO (Dobility, Inc.) by the two assessors. The two assessors’ scores were arithmetically averaged.

### 2.7 Data Analyses

Descriptive statistics were used to summarize baseline characteristics of the index and contact clients, as well as features of the counselling sessions. Descriptive analysis was conducted to calculate the duration of counselling from the timestamps. Missing data were infrequent and were excluded from analysis. To assess agreement [28] of the two assessors’ fidelity scores, we estimated Cohen's kappa with a corresponding 95% confidence interval (CI) constructed via percentile‐based bootstrap (1000 replications). Landis and Koch's threshold values were used to interpret Cohen's kappa results [29]. Kappa values range from 0 (absence of agreement) to 1 (perfect agreement). A Cohen's kappa value ≥0.81 was considered excellent agreement; from 0.61 to 0.80 good; from 0.41 to 0.60 moderate; from 0.21 to 0.40 fair; and ≤0.20 slight to be poor (Table S1). The Bland and Altman method [30] was used to compare the assessors’ scores. Two‐sample *t*‐tests were used to compare mean fidelity scores for each item on the checklist between the Standard and Enhanced arm.

Differences in fidelity scores with 95% CIs were compared between Standard and Enhanced arms using a general linear model fit using generalized estimating equations with an identity link and normal distribution and an independent working correlation matrix with robust sandwich‐type variance estimation to account for potential correlation between observations at the cluster level. Fidelity scores for index and contact clients were analysed separately. We applied a 0.05 type I error rate, and constructed 95% CIs without adjustment for multiplicity. Statistical power calculations were described previously [20]. Data cleaning and analyses were conducted in STATA (version 19.0).

### 2.8 Ethical Approval

The study was reviewed and approved by the Malawi National Health Sciences Research Committee, the University of North Carolina at Chapel Hill Institutional Review Board and the Baylor College of Medicine Institutional Review Board. Written informed consent was obtained from all study participants. The study was registered at clinicaltrials.gov (NCT05343390). Artificial intelligence tools were not used in the research and writing process.

## 3 Results

### 3.1 Population Characteristics

There were *N* = 33 clusters with 11 in the Enhanced arm and 22 in the Standard arm where index and contact clients were recruited (Figure 1).

**FIGURE 1:**
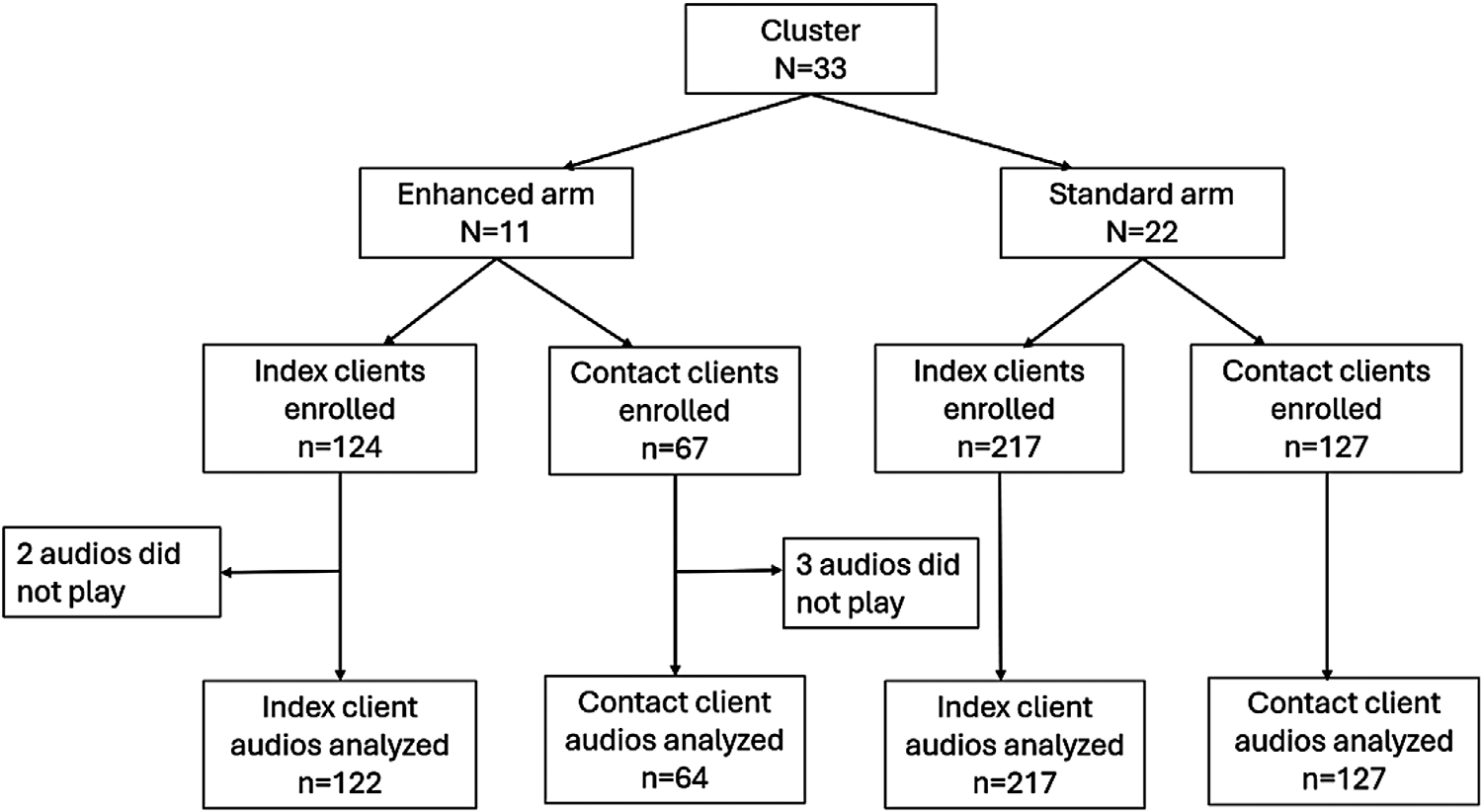
A flowchart of the index and contact clients enrolled in the study.

We enrolled 341 index clients, 124 (36.4%) in the Enhanced arm and 217 (63.6%) in the Standard arm. We enrolled 5–24 index clients per cluster. Median age was 34 years (IQR: 27–42). Most were female (68%), married (63%) and had less than secondary education (60%). More than half (53%) of the index clients were recruited from the HIV testing service setting.

We enrolled 194 contact clients, 67 (34.5%) in the Enhanced arm and 127 (65.5%) in the Standard arm. We enrolled 4–8 contact clients per cluster. The median age was 37 years (IQR: 28−47). A majority were male (56%), married (83%) and had less than secondary school education (57%). Most (64%) of the contact clients were recruited at the health facility (Table 1).

**TABLE 1:** Index and contact clients characteristics by study arm.

	Index clients		Contact client	
Characteristic, *n* (%)	Enhanced arm (*n* = 124)	Standard arm (*n* = 217)	Enhanced arm (*n* = 67)	Standard arm (*n* = 127)
Age (years)				
0–15	5 (4.0)	11 (5.1)	9 (13.4)	23 (18.1)
16–29	37 (29.8)	67 (30.9)	15 (22.4)	28 (22.1)
30–39	36 (29.0)	75 (34.6)	14 (20.9)	31 (24.4)
40–49	26 (21.0)	37 (17.1)	16 (23.9)	25 (19.7)
50+	20 (16.1)	27 (12.4)	13 (19.4)	20 (15.8)
Sex^a^				
Male	47 (37.9)	61 (28.1)	39 (67.2)	63 (60.0)
Female	77 (62.1)	156 (71.9)	19 (32.8)	42 (40.0)
Marital status^b^				
Never married	5 (4.2)	9 (4.4)	1 (1.7)	7 (6.7)
Married	73 (61.3)	131 (63.3)	52 (89.7)	88 (83.8)
Divorced or separated	32 (26.9)	55 (26.6)	4 (6.9)	9 (8.6)
Widowed	9 (7.6)	12 (5.8)	1 (1.0)	1 (1.7)
Education^b^				
No school	24 (20.2)	43 (20.8)	6 (10.3)	19 (18.1)
Some primary education	66 (55.5)	129 (62.3)	32 (55.2)	65 (61.9)
Completed primary	8 (6.7)	13 (6.3)	9 (15.5)	6 (5.7)
Some secondary or tertiary education	21 (17.6)	22 (10.6)	11 (19.0)	15 (14.2)
Able to read and write^b^				
No	53 (44.5)	89 (43.0)	16 (27.6)	46 (43.8)
Yes	66 (55.5)	118 (57.0)	42 (72.4)	59 (56.2)
Client type				
Adult client	119 (96.0)	207 (95.4)	58 (86.6)	104 (81.9)
Paediatric client	5 (4.0)	10 (4.6)	9 (13.4)	23 (18.1)
Recruitment venue				
ART clinic	55 (44.4)	104 (47.9)		
HIV testing service clinic	69 (55.6)	113 (52.1)		
Facility			42 (62.7)	83 (65.4)
Community			25 (37.3)	44 (34.6)
District				
Balaka	43 (34.7)	93 (42.9)	28 (41.8)	54 (42.5)
Machinga	81 (65.3)	124 (57.1)	39 (58.2)	73 (57.5)
Facility type				
District hospital	16 (12.9)	30 (13.8)	6 (9.0)	14 (11.0)
Health centre	94 (75.8)	175 (80.7)	55 (82.1)	101 (79.5)
Dispensary	14 (11.3)	12 (5.5)	6 (9.0)	12 (9.5)
Recent HIV test				
Date of survey	68 (55.7)	113 (52.1)	66 (98.5)	127 (100.0)
Before survey	55 (44.3)	104 (47.9)	1 (1.5)	0 (0.0)
Last HIV test result				
Negative	0 (0.0)	0 (0.0)	63 (94.0)	112 (88.2)
Positive	123 (100.0)	216 (99.5)	4 (6.0)	13 (10.2)
Indeterminate	0 (0.0)	1 (0.5)	0 (0.0)	2 (1.6)

Abbreviation: ART, antiretroviral therapy.

^a^ Missing sex for paediatric contact clients.

^b^ Missing marital status, education level and literacy level for paediatric clients.

The index client‐HCW encounters were 13.5 min (SD: 7.1) in the Enhanced arm and 10.3 min (SD: 7.9) in the Standard arm. The contact client‐HCW encounters audios were 22.3 min (SD: 10.3) in the Enhanced arm and 24.7 min (SD: 10.5) in the Standard arm.

### 3.2 Fidelity Scores Inter‐Assessor Reliability

For each item in the index client‐HCW encounters, inter‐assessor reliability with Cohen's kappa ranged from 0.43 to 0.94. Similarly, for the contact client‐HCW encounters, Cohen's kappa ranged from 0.58 to 0.99 (Table S1). The Bland and Altman plots demonstrated good agreement between the assessors (Figure S1a,b).

### 3.3 Effect of the Blended Learning Implementation Package on Intervention Fidelity

Fidelity scores were higher across each item in the Enhanced arm compared to the Standard arm (Figure 2a,b). For the index fidelity checklist (Figure 2a), there were statistically significant differences between 14 of 15 items. For the contact fidelity checklist (Figure 2b), there were significant differences between seven of 15 items. For the index‐HCW encounters, the technical content items improved more than the counselling quality, mean difference (24.4%; 95% CI: 19.4%, 29.4) compared to (15.8%; 95% CI: 12.4%, 19.2%). For the contact‐HCW encounters, the mean differences were similar for technical content (11.1%; 95% CI: 5.4%, 16.7%) and counselling quality (11.7%; 95 CI: 6.2%, 17.2%).

**FIGURE 2:**
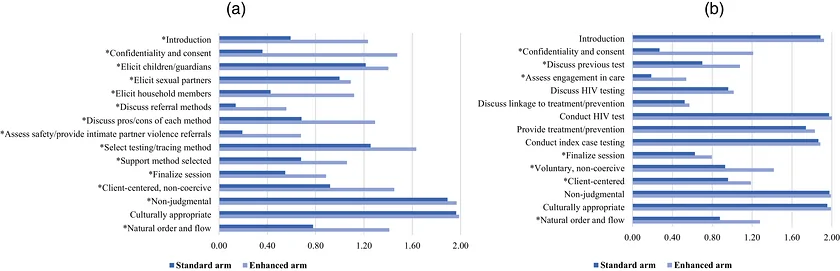
(a) Mean score for each index fidelity checklist item. (b) Mean score for each contact fidelity checklist item. *Denotes items that were significant using *t*‐test analysis.

The mean fidelity score for index client‐HCW encounters was higher in the Enhanced arm (66.3%; 95% CI: 62.8%, 69.7%) compared to the Standard arm (45.7%; 95% CI: 42.7%, 48.6%). The mean fidelity score difference was 20.6% (95% CI: 16.1%, 25.2%, *p*<0.001) (Figure 3). Comparable differences between arms were observed in the HIV testing setting (20.7%; 95% CI: 15.0%, 26.3%) and antiretroviral treatment setting (20.8%; 95% CI: 14.9%, 26.7%). Comparable differences between arms were also observed for male (23.0%; 95% CI: 15.6%, 30.3%) and female (19.6%; 95% CI: 15.7%, 23.5%) index clients.

**FIGURE 3:**
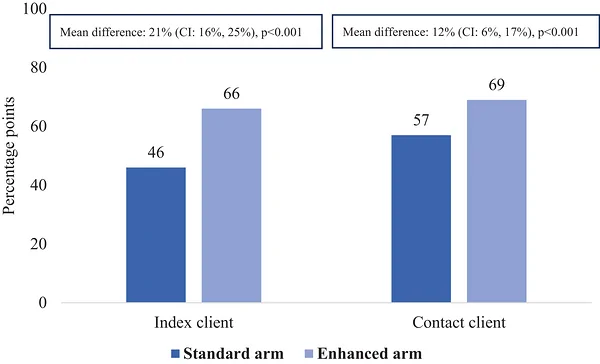
Health care worker fidelity to index case testing outcomes by study arm. Abbreviation: CI, confidence intervals.

The mean fidelity score for contact client‐HCW encounters was higher in the Enhanced arm (69.0%; 95% CI: 63.8%, 74.1%) compared to the Standard arm (57.4%; CI: 55.5, 59.3%). The mean fidelity score difference was 11.5% (CI: 6.1%, 17.1%, *p*<0.001). Comparable differences between arms were observed in the facility (13.6%; CI: 8.5%, 18.6%) and community (8.5%; CI: −0.3%, 17.3%) settings (Table 2). Comparable differences between arms were also observed for male (11.6%; 95% CI: 5.5%, 17.8%) and female (8.7%; 95% CI: 2.0%, 15.4%) contact clients.

**TABLE 2:** Health care worker fidelity to index case testing per setting and population by study arm.

Index clients	Setting/population	*n*	Enhanced arm Mean (95% CI)	Standard arm Mean (95% CI)	Mean difference (95% CI)
	HIV testing services	181	65.1 (60.8, 69.3)	44.4 (40.6, 48.2)	20.7 (15.0, 26.3)
	ART services	158	67.8 (63.5, 72.1)	47.0 (43.0, 51.1)	20.8 (14.9, 26.7)
	Adult	323	66.3 (63.1, 69.6)	45.4 (42.5, 48.3)	20.9 (16.6, 25.3)
	Paediatric	16	64.8 (49.7, 79.9)	50.1 (42.1, 58.0)	14.7 (−2.4, 31.8)
	**Overall**	**339**	**66.3 (62.8, 69.7)**	**45.7 (42.7, 48.6)**	**20.6 (16.1, 25.2)**

Contact clients					
	Facility	123	72.1 (67.5, 76.7)	58.6 (56.6, 60.6)	13.6 (8.5, 18.6)
	Community	68	63.7 (55.7, 71.7)	55.2 (51.6, 58.8)	8.5 (−0.3, 17.3)
	Adult	160	70.7 (64.9, 76.5)	57.6 (55.6, 59.7)	13.1 (6.9, 19.2)
	Paediatric	31	58.5 (53.3, 63.7)	56.3 (53.0, 59.6)	2.2 (−3.9, 8.4)
	**Overall**	**191**	**69.0 (63.8, 74.1)**	**57.4 (55.5, 59.3**)	**11.5 (6.1, 17.1)**

Abbreviations: ART, antiretroviral therapy; CI, confidence interval.

## 4 Discussion

We developed and evaluated a digital training implementation strategy to support fidelity to index case testing procedures in Malawi. The package improved lay HCWs’ fidelity to index and contact client counselling. This positive impact was observed across multiple settings and types of index and contact clients.

Our study is the first demonstrating a digitally guided training's ability to improve HCW *behaviour* in LMICs. Many trainings show positive changes in knowledge and attitudes but rarely impact HCWs’ skills and behaviours [16]. We believe the thoughtful development of the digitally guided training was critical to its effectiveness. Our training development was guided by formative research and the theory, emphasizing the role of learning, modelling, practicing and receiving feedback as key elements for the acquisition of robust skills. These activities have improved skills in medical education for professional HCWs [31] and counselling skills of lay HCWs [32] in similar settings. Our results support other findings from LMICs [15] demonstrating the success of training designed with these activities in building the capacity of HCWs. Therefore, we build on these findings by showing that these steps can be achieved through digital means.

The digital training had differential effects across items on the checklists—improving some items substantially more than others. This may be due to the health workers having prior HIV counselling and testing experience [33], resulting in the observed ceiling effect mainly on the contact fidelity checklist. Additionally, despite the significant improvement in fidelity in the Enhanced arm, health workers’ fidelity scores did not reach 100%, indicating that ongoing quality improvement could be needed.

Greater fidelity tends to yield more desirable clinical outcomes. In a separate analysis as part of PRACTICE, we evaluated clinical outcomes and found that digital training had an impact on contact clients elicited and tested [21]. Notably, in this current analysis, we found that fidelity to index case testing protocols was better in the digital training arm. This finding may suggest that fidelity could be a mechanism through which digital training improved clinical outcomes [21]. In addition, findings from this analysis substantiate other literature [26, 34] that argues that fidelity could moderate the relationship between an intervention and its main outcomes.

The rigorous nature of our study design is an important strength. First, we conducted a CRCT, which provided conditions to evaluate the digital training implementation strategy's effectiveness. Second, we had two blinded assessors evaluate the client‐HCW encounter audios, minimizing bias in the scoring and leading to credible results. Third, for the index and contact client data, we found that results for Cohen's kappa were moderate to almost perfect, as per Landis and Koch's scale, indicating a high level of inter‐assessor reliability. Furthermore, the Bland and Altman plot demonstrated high agreement between the assessors. The reliability results were concordant with the two assessors. These findings suggest that the fidelity scores can be interpreted as internally valid.

Implementation of interventions in real‐world settings cannot surpass the skill levels of the implementers and whether the intervention is implemented with integrity to the protocol. Despite the importance of fidelity in clinical practice, there is a scarcity of published literature assessing fidelity in behavioural randomized controlled trials [35, 36, 37]. Feely et al. [10] suggested that developing a reliable and valid measure of fidelity should receive the same level of attention as other aspects of the study. In PRACTICE, we were able to comprehensively assess fidelity through measuring adherence and quality, an important strength of the overall study. Our work serves as one model of how to integrate the fidelity evaluation in a real‐world setting.

Measuring fidelity is challenging. We conducted direct observation of the client‐HCW encounters, but the presence of research staff may have influenced HCW performance through the Hawthorne effect [38]. However, this is unlikely to explain the differences we observed between study arms due to the blinded nature of assessments and comparable procedures between arms.

Additionally, scoring fidelity items on an ordinal scale may not fully reflect the different levels of performance. Two scores of “1” may not be equal to one score of 2. However, we opted for a three‐level response option, rather than a binary response option, because it was common for HCW to have partial competence for a single step. For example, an HCW might introduce index case testing, but forget to introduce themselves. Or they might have a natural order and flow through much of the session, but not all of it. Capturing these partial achievements felt important, even if imperfect.

A related concern is that fidelity differences may have differed by the client's gender. To address this potential concern, we conducted stratified analyses and found that effects were comparable in male and female index and contact clients. We did not examine sex concordance between clients and health workers because this practice is not feasible in this setting. However, it is possible that the absence of sex concordance attenuated results.

Another limitation of our fidelity measures is the reliance on audio, rather than video. It is possible that this may have resulted in missed cues in body language or facial expressions. Future studies should consider video recording the client‐HCW encounters.

Contamination was a small potential risk in this study. We minimized this by controlling access to the training tablets, thereby reducing opportunities for HCWs in the Standard arm to access the digital training content. We also tracked all staff transfers from one facility to another, and this was minimal (<1% of health workers).

The results from this study are timely and contribute new knowledge on how to train HCWs. Most countries have adopted index case testing as policy [15, 39]. Digital training approaches could be leveraged for capacity‐building for the HCWs’ service delivery programmes. Notably, these training approaches have the potential to be adapted in different geographical settings and cultures, and benefit other index testing programmes, including sexually transmitted infections and hepatitis B. Our study was conducted before the current shifts in policy and reduction of funding and ensuing recommendations for budget‐friendly training programmes, which have made large in‐person trainings limited. As such, these digital training approaches could be ideal for various remote training needs, which are now even more imperative than before. Digital training programmes hold the potential to train different types of HCWs and could be convenient, feasible and scalable for building the capacity of HCWs when compared to centralized in‐person training, which is supported by literature and reviews from LMICs [40, 41, 42].

## 5 Conclusions

The digital training implementation package led to improvements in HCWs’ counselling behaviours and ultimately in clinical outcomes. The lessons learned and findings from our study offer a promising approach for bringing effective index case testing interventions to scale, and improving timely access to HIV testing, treatment and prevention services, in support of UNAIDS’ 95–95–95 goals by 2030.

## Author Contributions

**Tapiwa A. Tembo**: Conceptualization, investigation, funding acquisition, Writing – original draft, methodology, validation, Writing – review and editing, formal analysis, supervision, project administration, visualization, resources. **Nora E. Rosenberg**: Conceptualization, investigation, funding acquisition, writing – review and editing, supervision, methodology, validation, visualization, resources. **Katie R. Mollan**: Conceptualization, investigation, writing – review and editing, methodology, validation, visualization. **Mike J. Chitani**: Data curation. **Mtisunge Mphande**: Data curation. **Jiayu Wang**: Data curation, writing – review and editing. **Victor Mwapasa**: Conceptualization, methodology, writing – review and editing. **Katherine Simon**: Conceptualization, investigation, writing – review and editing. **Saeed Ahmed**: Conceptualization, writing – review and editing, resources. **Maria H. Kim**: Conceptualization, investigation, methodology, writing – review and editing. **Linda‐Gail Bekker**: Conceptualization, investigation, methodology, validation, writing – review and editing, supervision.

## Funding

Research was supported by R01 MH124526. TAT was supported by the Fogarty International Center of the National Institutes of Health under Award Number D43 TW010060. MHK was supported by the National Institutes of Health under R01 MH115793‐04. KRM and NER were supported in 2019 by P30 AI050410. The funders had no role in trial design, data collection and analysis, decision to publish or preparation of the manuscript.

## Conflicts of Interest

The authors declare no conflicts of interest.

## Supporting information




**Supplementary Table 1**: Cohen's kappa values by two assessors for index and contact client fidelity scores,
**Supplementary Information 1**: Fidelity Assessment Checklists,
**Supplementary Figure 1a**: Bland and Altman plot of index clients‐HCW fidelity score, etc.,
**Supplementary Figure 1b**: Bland and Altman plot of contact clients‐HCW fidelity scores.

## Data Availability

The data that support the findings of this study are available from the corresponding author upon reasonable request.
