# Supplementary material for: Assessing Fidelity to HIV Index Case Testing Protocols in Malawian Clinical Settings: Results From a Cluster Randomized Controlled Trial
Source: J Int AIDS Soc. 2026 Oct 8;29(Suppl 5):e70185. doi: 10.1002/jia2.70185 (PMC13650527; doi:10.1002/jia2.70185)
Supplement: Supplementary file 1 [file JIA2-29-e70185-s001.docx]

**Supplementary Tables**

***Supplementary* Table 1: Cohen’s kappa values by two assessors for index and contact client fidelity scores**

| **Item** | **Raters** | **Kappa value (95% CI)** | **Agreement** |
| --- | --- | --- | --- |
| **Index client checklist** | | | |
| Introduction | 2 | 0.87 (0.82, 0.91) | 90.0% |
| Confidentiality and consent | 2 | 0.94 (0.77, 1.00) | 95.9% |
| Elicit children/guardians | 2 | 0.89 (0.84, 0.92) | 90.6% |
| Elicit sexual partners | 2 | 0.85 (0.75, 0.92) | 95.9% |
| Elicit household members | 2 | 0.68 (0.62, 0.75) | 71.7% |
| Discuss referral methods | 2 | 0.80 (0.66, 0.91) | 92.9% |
| Discuss pros/cons of each method | 2 | 0.69 (0.62, 0.75) | 74.9% |
| Assess safety/provide IPV referrals | 2 | 0.66 (0.54, 0.76) | 87.0% |
| Select testing/tracing method | 2 | 0.61 (0.51, 0.70) | 74.3% |
| Support method selected | 2 | 0.75 (0.68, 0.81) | 81.1% |
| Finalize session | 2 | 0.49 (0.40, 0.59) | 81.4% |
| Client-centered, non-coercive | 2 | 0.67 (0.60, 0.75) | 76.1% |
| Non-judgmental | 2 | 0.43 (0.28, 0.58) | 91.7% |
| Culturally appropriate | 2 | 0.62 (0.36, 0.88) | 97.9% |
| Natural order and flow | 2 | 0.70 (0.62, 0.76) | 77.0% |
| **Contact client checklist** | | | |
| Introduction | 2 | 0.84 (0.58, 1.00) | 98.8% |
| Confidentiality and consent | 2 | 0.91 (0.85, 0.95) | 97.4% |
| Discuss previous test | 2 | 0.86 (0.78, 0.93) | 93.7% |
| Assess engagement in care | 2 | 0.89 (0.58, 1.00) | 97.3% |
| Discuss HIV testing | 2 | 0.73 (0.56, 0.86) | 98.4% |
| Discuss linkage to treatment/prevention | 2 | 0.83 (0.76, 0.90) | 97.8% |
| Conduct HIV test | 2 | 0.85 (0.57, 1.00) | 99.5% |
| Provide treatment/prevention | 2 | 0.91 (0.81, 0.97) | 98.2% |
| Conduct index case testing | 2 | 0.99 (0.94, 1.00) | 99.9% |
| Finalize session | 2 | 0.64 (0.52, 0.76) | 95.9% |
| Voluntary, non-coercive | 2 | 0.62 (0.51, 0.75) | 95.9% |
| Client-centered | 2 | 0.58 (0.47, 0.67) | 93.9% |
| Non-judgmental | 2 | 0.72 (non-convergence) | 99.6% |
| Culturally appropriate | 2 | 0.90 (non-convergence) | 99.7% |
| Natural order and flow | 2 | 0.82 (0.72, 0.90) | 97.0% |

NB: Agreement was part of the output from the Kappa values calculation

**Supplementary information 1: Fidelity Assessment Checklists**

**Index Clients Fidelity Checklist**

Instructions: For each item, please check only one response. If something is not applicable, check NA.

| **During the counseling session, did the counselor…?** | |  | |  |  |  | |
| --- | --- | --- | --- | --- | --- | --- | --- |
| **Counseling Content** | | **Needs improvement** | **Satisfactory** | | **Excellent** | | **Not applicable** |
| **Stage 1: Explore** | | | | | | | |
| 1 | Introduce the session appropriately.   - Introduce yourself, welcome the client and build rapport. - Explain the purpose of the conversation. - Explain that the process is confidential |  |  | |  | |  |
| 2 | Introduce key counselling principles appropriately.   - Obtain verbal consent to proceed |  |  | |  | |  |
| 3 | >=15 years: Ask about biological children and determine which need testing and/or linkage to care OR  <=14 years: Ask about parents/guardians and determine which need testing and/or linkage to care |  |  | |  | |  |
| 4 | >=15 years: Ask about sexual partners and determine which need testing and/or linkage to care |  |  | |  | |  |
| 5 | Ask about household members and social contacts and determine which need testing and/or linkage to care |  |  | |  | |  |
| **Stage 2: Understand** | | | | | | | |
| 6 | Appropriately assess safety and risk of IPV and provide referrals as needed |  |  | |  | |  |
| 7 | Discuss the different methods of ICT: Passive referral, Passive referral with STK, Contract referral, Provider referral, and Dual referral) |  |  | |  | |  |
| 8 | Help the client understand the pros and cons of each option and the barriers and facilitators |  |  | |  | |  |
| 9 | Help the client select an appropriate referral method for each contact |  |  | |  | |  |
| **Stage 3: Act** | | | | | | | |
| 10 | Adequately support the method(s) selected:  Passive: FRS (and STK) provided and explained  Contract/Provider: Contact information obtained, and plan discussed.  Dual: Details of couple testing provided |  |  | |  | |  |
| 11 | Finalize the session   - Summarize action items (who will be tested with which method) - Give the client an opportunity to ask questions |  |  | |  | |  |
| **Counseling Quality** | | | | | | | |
| 12 | Was the conversation client-centered and non-coercive? |  |  | |  | |  |
| 13 | Did the counselor demonstrate non-judgmental attitudes towards the client? |  |  | |  | |  |
| 14 | Was the session conducted in a way that was culturally and linguistically appropriate? |  |  | |  | |  |
| 15 | Did the session have a natural order and flow? |  |  | |  | |  |

**Contact Clients Fidelity Checklist**

Instructions: For each item, please select only one response. If something is not applicable, check NA.

| **During the counseling session, did the counselor…?** | |  |  |  | |  |
| --- | --- | --- | --- | --- | --- | --- |
| **Counseling content** | | **Needs improvement** | **Satisfactory** | | **Excellent** | **Not applicable** |
| **Stage 1: Explore `** | | | | | | |
| 1 | Introduce the session appropriately   - Make sure you are speaking to the correct person. - Introduce yourself and build rapport. - Explain the purpose of the conversation |  |  | |  |  |
| 2 | Introduce counselling approach:   - Explain that the process is confidential. - Obtain verbal consent to proceed |  |  | |  |  |
| 3 | Discuss timing and results of contact’s last test. |  |  | |  |  |
| 4 | If HIV-positive, assess engagement in treatment.  If HIV-negative, assess engagement in prevention. |  |  | |  |  |
| **Stage 2: Understand** | | | | | | |
| 5 | If needed, discuss reasons to test; address barriers; and discuss facilitators.  Offer same day rapid testing (opt-out), referral, or self-test kits. |  |  | |  |  |
| 6 | If needed, discuss linkage to prevention or treatment. Address barriers and discuss facilitators. |  |  | |  |  |
| **Stage 3: Act** | | | | | | |
| 7 | Support/conduct the testing method(s) selected:   - Same day rapid test - Self-test kit - Referral for rapid test |  |  | |  |  |
| 8 | Provide treatment or prevention referrals as needed. |  |  | |  |  |
| 9 | Determine whether client is also an index and needs index counseling. |  |  | |  |  |
| 10 | Finalize the session   - Summarize action items. - Give the contact an opportunity to ask questions |  |  | |  |  |
| **Counseling Quality** | | | | | | |
| 11 | Was the conversation voluntary and non-coercive? |  |  | |  |  |
| 12 | Was the conversation client-centered? |  |  | |  |  |
| 13 | Did the counselor demonstrate non-judgmental attitudes towards the client? |  |  | |  |  |
| 14 | Was the session conducted in a way that was culturally and linguistically appropriate? |  |  | |  |  |
| 15 | Did the session have a natural and logical order and flow? |  |  | |  |  |

***Supplementary* Figure 1a: Bland and Altman plot of index clients-HCW fidelity score**

***Supplementary* Figure 1b: Bland and Altman plot of contact clients-HCW fidelity scores**
